# An Augmented Full-Body Model that Improves Upper Body Tracking and Reduces Dynamic Inconsistency in Complex Motion

**DOI:** 10.1007/s10439-025-03762-7

**Published:** 2025-06-03

**Authors:** Xiao Hu, Evan A. Dooley, Darren J. Stefanyshyn, John W. Wannop, Shawn D. Russell

**Affiliations:** 1 Department of Biomedical Engineering, University of Virginia, Charlottesville, VA USA; 2 Department of Mechanical and Aerospace Engineering, University of Virginia, Charlottesville, VA USA; 3 Human Performance Lab, Faculty of Kinesiology, University of Calgary, Calgary, Canada; 4 Department of Orthopedic Surgery, University of Virginia, Charlottesville, VA USA

**Keywords:** Full-body musculoskeletal model, Inverse kinematics, Inverse dynamics, Marker tracking

## Abstract

**Purpose:**

In recent years, the applications of musculoskeletal simulations have been expanded from simple walking to complex movements in various kinds of sports. The goal of this study was to augment the capability of the currently widely used full-body model (Rajagopal (2016) IEEE Trans. Biomed. Eng. 63:2068–2079) to improve the tracking of the kinematics of the head, shoulder, arms, and torso during complex full-body motion.

**Methods:**

Based on the testing of different modeling choices of neck, shoulder, and torso segments, the original Rajagopal full-body model was augmented by adding three joints in the spine and two sternoclavicular joints. The inverse kinematics and inverse dynamics of sports-related movements from 16 collegiate athletes were compared between the original Rajagopal and augmented full-body model.

**Results:**

Our results showed that the augmented full-body model had significant improvements in tracking errors of the markers on the head, arm, torso, and pelvis during inverse kinematics, which led to reduced dynamic inconsistency in inverse dynamics, compared to the Rajagopal model.

**Conclusion:**

With a significant improvement in tracking the kinematics of the upper body, the augmented full-body model is a more suitable model to perform simulations involving complex full-body movements and is available for research use upon request from simtk.org.

## Introduction

In the past 30 years, because of its unique capability of providing estimates about quantities (e.g., joint contact force, muscle forces, and length changes) that are otherwise hard to measure directly, musculoskeletal modeling and simulations have been widely used in understanding the neural control and biomechanics of human movement, revealing pathological mechanisms of movement disorders and identifying injury mechanisms in various types of sports [1, 2]. One of the initial focuses of musculoskeletal simulations has been the walking motion, which is crucially related to our health and daily mobility [3–6]. Since the walking motion primarily involves cyclic movements of the lower limbs primarily in the sagittal plane with little motion in the upper body, musculoskeletal models with lower limbs alone or with a simplified upper body (i.e., the head, arms and torso lumped into a single rigid body) [3, 7–9] were sufficient to capture the main biomechanics of walking, which greatly advanced our understanding of basic locomotion strategies. In recent years, the applications of musculoskeletal simulations have been expanded to various types of sports, some of which involve complex motion with large bending, listing and rotation of upper body, to identify and assess risk factors for related injuries [10–16]. Although the most common injuries during practice and competitions happen in lower limbs in sports [17], the lower limb model alone or with a simplified upper body may not be sufficient and thus affect the efficacy of the simulations of sports-related movements due to the coordinated motion of various body segments involved in these complex movements.

To better capture the movements of not only the lower limbs but also the torso, shoulder, neck, and head in complex, coordinated movements, there have been efforts to develop full-body musculoskeletal models. Hamner, Seth [18] and Rajagopal, Dembia [19] expanded the previous versions of the lower limb models [3, 7, 8] to include the arms, shoulders, torso, and head, therefore creating a full-body model. However, the shoulders, head, and torso of this model are lumped as one rigid segment connected to the pelvis by a ball-and-socket joint, essentially allowing no independent movements of these body segments. Previous studies also have combined existing spine models [20, 21] with lower limb models [3, 7, 8] to develop full-body models that better characterize the torso movements. However, Actis, Honegger [22] primarily looked into the planar trunk-pelvis motion without including head, neck, and arms in the model; Raabe and Chaudhari [23] lumped the shoulder girdle, neck, and head into the torso when investigating jogging biomechanics; Burkhart, Grindle [24] and Favier, Finnegan [25] mainly focused on the movements of the spine and both did not incorporate shoulder girdle movement. Cazzola, Holsgrove [11] focused on capturing the neck and shoulder girdle movements by incorporating an existing neck model [26] and a scapula-clavicular joint into an existing lower limb model [7, 27]. However, the musculature of the lower limb was not included and the whole torso was simplified to one segment. Caruthers, Thompson [28] incorporated a lower limb model[8], a lumbar spine model [21], an upper limb model [29], and a neck model [26] to develop a full-body model when examining the sit-to-stand transfer. However, the incorporated lumbar spine model may not be suited for movement involving large torso flexions. In addition, some of the full-body models included many musculotendon actuators to represent torso muscles and joints among vertebrae and ribs [23–25]. Although these elements are needed for investigating spinal loadings, the increased complexity of the model may unnecessarily reduce the speed of simulations if the goal of the simulation is to examine the functions of the lower limb. The above discussion may not have included all the full-body models in the literature, but it is clear that a full-body model with emphasis on the lower limb and capable of tracking the upper body is needed for the field to expand the use of musculoskeletal modeling and simulations to various types of sports.

The goal of this work was to augment the capability of the currently most widely used full-body model [19] to improve the tracking of the kinematics of the head, shoulder, arms, and torso during complex/coordinated full-body motions, while maintaining the complexity of the model to mainly focus on the lower limb musculature. We achieved this goal by adding 3 joints in the spine and 2 joints between clavicles and sternum based on the existing models of various body segments [26, 29]. We hypothesized that the better characterization of the kinematics of the upper body in the augmented model will improve the overall tracking errors in inverse kinematics compared with the original model [19] and that the improvement in inverse kinematics will reduce the dynamic inconsistency in inverse dynamics. We tested these hypotheses by comparing the inverse kinematics and inverse dynamics from movements often involved in various sports from 16 collegiate athletes based on the augmented full-body model vs. the original full-body model [19]. These comparisons showed a significant improvement in tracking the kinematics of the upper body, which then led to reduced dynamic inconsistency in inverse dynamics in the augmented full-body model. This augmented full-body model was implemented in OpenSim [1] and is freely available for research use through https://simtk.org/projects/sports_model/ upon request.

## Methods

To augment the Rajagopal full-body model [19], we first tested and compared the various models of neck, shoulder, and torso segments as detailed below. Then, we augmented the original Rajagopal full-body model with the tested segment models that improved tracking accuracy without significantly adding to the overall complexity of the full-body model. All model development and testing were done in OpenSim 4.4 [1].

### Tested Neck Models

**Fig. 1:**
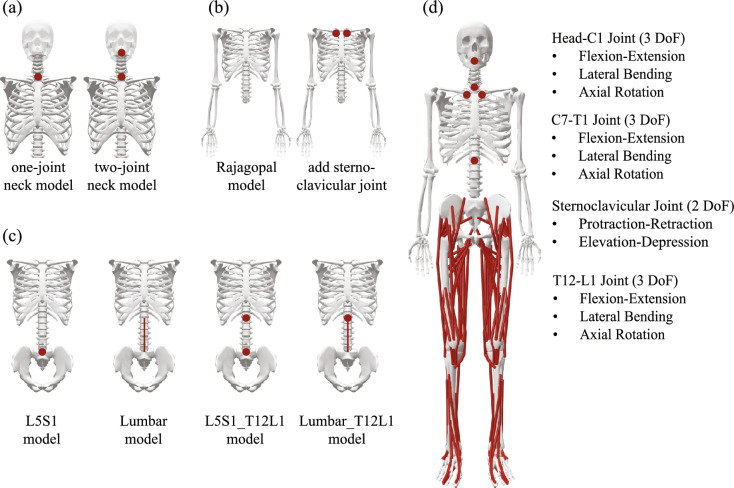
The illustrations of the **a** tested necked models, **b** tested shoulder models, **c** tested torso models, and **d** the additional joints implemented in the augmented full-body model. These joints connect the rigid bodies, including abdomen, thorax, neck, head, and clavicles (right and left), split from the lumped upper body segment in the original Rajagopal model [19]

The original Rajagopal full-body model [19] does not include any joint in the cervical spine, which prevents any meaningful tracking of the head motion. To improve the tracking of the head motion, two versions of the neck models were tested (Fig. 1a). The first one was created by adding a custom joint with three rotational degrees of freedom (DoF; assuming negligible translations) between the seventh cervical vertebra (C7) and first thoracic vertebra (T1) to the Rajagopal model, which will be referred as the one-joint neck model. The second one was created by adding a custom joint with three rotational DoFs between C7 and T1 and at the top of the first cervical vertebra (C1), which reflects possible independent motion of the upper and lower cervical spine [26] and will be referred as the two-joint neck model. In the two-joint neck model, the total axial rotation of the head relative to the torso was evenly distributed between these two joints in the cervical spine. The center of rotation of the joint between C7 and T1 is at the posterior border of the vertebral endplates and the anterior border of the spinal canal [30] and that of the joint between C1 and the skull is at the top of C1 is based on [11].

### Tested Shoulder Models

The original Rajagopal full-body model [19] only had a ball-and-socket joint as the glenohumeral joint in the shoulder girdle, which may limit the tracking of the shoulder motion. To improve the tracking of the shoulder motion, the sternoclavicular joints, which were modeled to have two rotational degrees of freedom to enable protraction–retraction and elevation–depression of the clavicle, were added to the original Rajagopal model to connect the clavicles of both sides to the torso (Fig. 1b). The scapulae were welded to the clavicles at the acromioclavicular joint. The joint locations and axes of rotations were defined as in [29], which followed [31]. Specifically, the sternoclavicular joint originates at the sternal end of the clavicle with its x-axis points from sternoclavicular joint to acromioclavicular joint, y-axis (protraction–retraction) perpendicular to the plane formed by its x and z axes, and z-axis (elevation–depression) perpendicular to the plane formed by its x-axis and the global axis pointing upward vertically. The acromioclavicular joint originates at the acromial end of the clavicle. The definition of the glenohumeral joint was kept as in the original Rajagopal full-body model [19].

### Tested Torso Models

The original Rajagopal full-body model [19] only had a custom joint with three rotational DoFs between first sacral vertebra (S1) and fifth lumbar vertebra (L5) and simplified the whole torso, neck, and head as a single segment connected to the pelvis by this joint, which may limit the tracking of the torso motion. To test whether the coupled motion from lumbar vertebrae as described in [21] and whether modeling the torso as thorax and abdomen segments (as opposed to a single segment) [32] may improve the tracking of torso motion, four versions of the torso models were tested (Fig. 1c).

The first tested torso model replicated the torso in the original Rajagopal model [19] by locking all the custom joints among lumbar vertebrae only except for the one between L5 and S1 vertebrae. This model will be referred as the L5S1 model. The second tested torso model was essentially the model developed by Christophy, Senan [21]. Briefly, this model includes a rigid pelvis and sacrum, the five lumbar vertebrae, and a rigid thorax consisting of a lumped thoracic spine and ribcage. The total lumbar motion (flexion–extension, lateral bending, and axial rotation) is distributed across five lumbar vertebrae with linear kinematic coordinate coupling constraints and predefined sets of ratios [21]. This model will be referred as the Lumbar model. The third tested torso model was built upon the first model and added one custom joint with 3 rotational DoFs between the 1st lumbar vertebra (L1) and the 12th thoracic vertebra (T12) to model the torso as two rigid bodies: the thorax and the abdomen [32]. This model will be referred as L5S1_T12L1 model. The fourth tested torso model was built upon the second model and added one custom joint with three rotational DoFs between the L1 and the T12 vertebra, essentially having an independent thorax from the coupled lumbar spine that forms abdomen. This model will be referred as the Lumbar_T12L1 model. In the L5S1_T12L1 and Lumbar_T12L1 models, the total axial rotation of the thorax relative to the pelvis was evenly distributed between the axial rotations of the thorax relative to abdomen and abdomen to pelvis.

### The Augmented Full-Body Musculoskeletal Model

Based on the testing and comparison of various models of neck, shoulder, and torso segments (see Results), the generic full-body musculoskeletal model [19] (reflecting a 75 kg, 170 cm tall male) was augmented to incorporate individual upper body segments that represent the coordinated movements of different segments during complex movements (Fig. 1d). The augmented model had a total of 29 articulating rigid bodies and 48 degrees of freedom (DoF). The 21 rigid bodies in the pelvis, upper limbs and lower limbs were inherited from the Rajagopal model. The augmented model also inherited 20 DoFs in the lower body (6 for the pelvis and 7 for each lower limb) and 14 DoFs (7 for each upper limb) in the upper body as well as 80 muscle-tendon units (40 per leg) to represent the musculature in the lower body from the Rajagopal model.

The main augmentations were in the upper body, where the lumped upper body segment in the Rajagopal model was split into eight rigid bodies, including abdomen, thorax, neck, head, clavicles (right and left), and scapulae (right and left). The torso was split into abdomen and thorax [32]. The abdomen encompasses the portion of the lumbar spine and the thorax encompasses the portion of the thoracic spine. They were connected to the pelvis as in the L5S1_T12L1 torso model tested above. The two-joint neck model was used to connect the thorax to the neck and then to the head segments. The shoulder girdle includes the clavicle and scapula welded together at the acromioclavicular joint and the sternoclavicular joint (two rotational DoFs) that connect the shoulder to the thorax, as the tested shoulder model.

The inertial properties (mass, center of mass and moment of inertia) of the augmented model were determined to maintain the overall mass (75 kg) and height (170 cm) of the original Rajagopal full-body model [19]. The pelvis, lower limbs and upper limbs of the augmented model inherited the same inertial properties from the Rajagopal model. The inertial properties of the abdomen, thorax, neck and head were calculated based on previous anthropometric studies about these body segments [32, 33]. Those of the clavicle and scapula were obtained from Ref. [29].

### Experimental Protocol

Sixteen collegiate athletes provided informed consent to participate in the IRB-approved protocol. These athletes were classified into two groups: (1) nine speed athletes (mass = 87.9 ± 11 kg, height = 184 ± 9 cm, age = 22.2 ± 2 years), who are specialized in speed and agility on the field (i.e., offense), and (2) seven power athletes (mass = 121.5 ± 17 kg, height = 192 ± 5 cm, age = 19.6 ± 2 years), who are specialized in strength on the field (i.e., defense). All athletes performed three movements: Agility, Vcut, and 3 Cone Cut. Group-specific movements were also performed: speed athletes performed Quick Stop and Drop Step movements, while power athletes performed Side Shuffle movement (see Table 1 for the details of each movement). For all movements, retro-reflective markers were placed over the entire body of athletes. The marker set of 57 markers consisted of a modified Plug-in Gait model with 9 additional markers on each lower limb for better tracking of the lower limbs (Table 2). Note that the marker set used here had a similar number of markers to that included in the original Rajagopal model but does not represent the minimum number of markers or the only marker set required for motion capture with the augmented model. Users may choose to add or remove certain markers based on the specific needs of their motion capture. The trajectories of the markers were recorded using a fifteen-camera high-speed motion capture system operating at 240 Hz (Vicon, Oxford Metrics, Oxfordshire, ENG). During each movement, ground reaction forces of the stance foot were collected at 2400 Hz using a Kistler force plate (Kistler AG, Winterthur, Switzerland) mounted in the center of a lab runway floor.

**Table 1:** The description of the movements performed in this study

Movement	Description
Agility	Each athlete ran 5 yards forward, planted right foot on the force plate, turn 180°, and then returned 5 yards to the starting position as quickly as possible.
Vcut	Each athlete ran 5 yards forward, planted his right foot on the force plate, and performed a 90° cut to his left-hand side, with the change in direction occurring on the force plate.
3 Cone Cut	Each athlete ran 5 yards forward, planted his right foot on the force plate, and performed a 90° cut to his right-hand side, with the change in direction occurring on the force plate.
Quick Stop	Each athlete ran 5 yards forward, planted his right foot in the center of the force platform, and then backpedaled out.
Side Shuffle	Each athlete side shuffled three steps to his right, planted his right foot on the force plate and reverse direction as quickly as possible, while facing the same forward direction.

**Table 2:** Description of the 57 markers used to track athletes during movements

Group	Marker(s)	Description
Head 4	LFHD, RFHD, LBHD, RBHD	Four markers around the top of the head, starting with left and right temple, should create level plane with ground when subject is looking straight ahead
Arms (L/R) 7/7	SHO	Shoulder, on the acromioclavicular joint
	UPA	Upper arm, on lateral upper arm
	ELB	Elbow, humeral lateral epicondyle
	FRM	Forearm, central posterior forearm in neutral rotates to aligned with WRB and ELB when palms face posterior
	WRA	Anterior wrist, radial styloid process
	WRB	Posterior wrist, ulnar styloid process
	FIN	Finger, distal 2nd metacarpal
Torso 5	C7	7th cervical vertebrae, base of neck
	T10	10th thoracic, on spine, usually about halfway down the back, where the ribcage comes together
	RBAK	Right back, somewhere on the right scapula, used so computer can detect right from left
	CLAV	Clavicle, jugular notch where clavicle meets sternum
	STRN	Sternum, on the xiphoid process of the sternum
Pelvis 4	LPSI, RPSI	Posterior superior iliac spine of pelvis, left and right side
	LASI, RASI	Anterior superior iliac spine of pelvis, left and right side
Leg (L/R) 15/15	THI1, THI2	Two markers on line between lateral knee and trochanter (THI1 superior to THI2)
	THIA	Marker on anterior side of thigh
	KNE	Lateral knee marker on lateral tibial plateau
	MKNE	Medial knee marker on medial tibial plateau
	TTUB	Tibial tuberosity
	HFIB	Proximal head of fibula
	SHN	Anterior tibia, midway between knee and ankle
	TIB	Lateral shank on line between lateral ankle and lateral knee
	ANK	Lateral malleolus
	MMA	Medial malleolus
	MPCT	Medial process of calcaneal tuberosity, Shoe = in corner of outer plastic heel cup on medial side
	LPCT	Lateral process of calcaneal tuberosity, Shoe = in corner of outer plastic heel cup on lateral side
	D1M	Distal 1st metatarsal
	D5M	Distal 5th metatarsal

### Marker Tracking Test with Inverse Kinematics

All the tested generic models were first scaled to match the anthropometry of the athletes [1]. Then, the Inverse Kinematics Tool in OpenSim was used to solve for the joint angles that minimized the tracking errors between the experimentally measured marker positions and the corresponding virtual markers on the models.

When testing each of the neck, shoulder, and torso models, a pair of base and tested segments were identified and isolated from other body segments to evaluate the effect of the model choices on the tracking performance. During inverse kinematics, the weights of marker groups on the base segment were set at 10 to ensure that the base segment was tracked equally well across different body segment models, whereas the weights of the marker groups on the tested segment were set at 1, so the differences in the tracking of the group of markers on the tested segment were due to the modeling choices that connected the base and the tested segments (Table 3, for the specific markers in each marker group, please refer to Table 2).

**Table 3:** The base and tested segments as well as the tracking weights of groups of markers during inverse kinematics for testing neck, shoulder, and torso models

	Base segment	Tested segment	Marker group weighted at 1	Marker group weighted at 10
Neck models	Torso	Head, neck	Head	Torso
Shoulder models	Torso	Arms	Arm	Torso
Torso models	Pelvis	Abdomen, thorax	Torso	Pelvis

When testing the full-body models (Rajagopal vs. the augmented models), tracking weights of all markers were set at 1, so the tracking errors of markers were counted equally in various body segments. All inverse kinematics were performed for the time period of the right stance phase plus 10% of the stance phase before the foot strike, in which the data had the best availability of markers. In total, 1155 inverse kinematics trials were run across 16 athletes to compare the tracking performance.

### Dynamic Inconsistency Test with Inverse Dynamics

Inverse dynamics with musculoskeletal models calculate the net forces and moments applied to the model that produce given motion. Because of the experimental errors and modeling assumptions, there always are dynamic inconsistencies between the model kinematics estimated from inverse kinematics and the measured ground reaction forces and moments [1, 34]. In the OpenSim, these dynamics inconsistencies are expressed as three residual forces and three residual moments applied to the three translational and three rotational DoFs of the model segment (pelvis in both full-body models) that connects the model to the ground. Since modeling errors contribute to the overall dynamic inconsistencies [35], better characterization of the upper body kinematics in the augmented full-body model may reduce the residual forces and moments at the pelvis.

The Inverse Dynamics Tool in OpenSim was used to calculate the required net joint moments and the residual forces and moments for the movements obtained from inverse kinematics (see Experimental Protocol and Model Testing with Inverse Kinematics above) and the associated ground reaction forces using the Rajagopal and the augmented full-body models. As a type of simulation involving no muscle, inverse dynamics simulations may not encounter the issue of insufficient muscle strength. Therefore, no reserve actuators were used. The movements were filtered at 20 Hz with the built-in low-pass filter in the Inverse Dynamics Tool. Two types of movements (3 Cone and VCut) were chosen, since they had single-support stance phases of the right leg (i.e., left foot was in the air), of which ground reaction forces and moments were recorded by a force plate. The inverse dynamics were performed during the stance phase of the right pivot foot when athletes were making a 90° cut to his right-hand side (3 Cone) or a 90° cut to his left-hand side (VCut). The vertical distances of 110 mm from the D1M and D5M markers of the left foot to the ground were used as a threshold to pick the trials in which the left foot was in the air during the entirety of the right stance phase. With this criterion, 94 out of 468 Vcut and 3 Cone Cut trials were identified across 14 athletes.

### Data Processing and Statistics

After inverse kinematics simulations were completed, the locations of the virtual markers on the model were recorded at each time frame of the simulations. Then, the root-mean-square errors (RMSE) between each virtual marker and its corresponding actual marker on the subjects were calculated across all the time frames of each inverse kinematic simulation. All 57 markers were divided into five groups: head, arm, torso, pelvis, and leg marker groups (Table 2). The averages of the RMSE were calculated across the markers in each of the five groups as the overall measures of the tracking performance of parts of the body during inverse kinematics simulations.

Ideally, in the absence of experimental and modeling errors, the residual forces and moments applied at the pelvis in the Rajagopal and augmented full-body models should be zero [1]. Thus, the root-mean-square (RMS) values of each of the residual forces and moments, which indicate the level of deviation of these quantities from zero, are calculated for each inverse dynamic simulation trial performed with the Rajagopal and augmented full-body models. Then, the ratio of the RMS from the augmented full-body model over that from the Rajagopal full-body model of each trial was calculated. A ratio smaller than 1 would indicate that the residual force/moment in the augmented full-body model is smaller than that from the Rajagopal full-body model, suggesting reduced dynamic inconsistency.

Linear mixed-effects (LME) models [36, 37] with model types and movement types as fixed effects and athletes as a random effect were used to examine the effects of model types on tracking performance (marker errors) during inverse kinematics. For the whole-body inverse kinematics simulations, separate LME models were constructed for each of the five markers groups. The specific null hypothesis tested by these LME models was that the model types (augmented model vs. original Rajagopal model) had no significant effect on the tracking performance, with the alternative hypothesis being model types had a significant effect. When a significant fixed effect of model type was revealed among tested torso models, post hoc comparisons were performed between the L5S1_T12L1 and the other three models. The LME models were also used with athletes as a random effect to assess the null hypothesis that the ratios of the RMS of residual forces and moments from inverse dynamics between augmented and Rajagopal full-body models were not different from 1 vs. the alternative hypothesis that the ratios were smaller than 1, indicating the augmented model had smaller/less error than the original Rajagopal model [19]. Visual inspection of the normal probability plot and parametric test of normality (i.e., the Shapiro–Wilk test [38]) did not reveal any obvious deviations from normality, indicating the normality assumptions were satisfied for the used LME models. All statistical analyses were conducted in Matlab (R2022b, Mathworks, Natick, MA, United States) with the significance level set at *p* < 0.05 and a power greater than 80% at the same significance level.

## Results

**Fig. 2:**
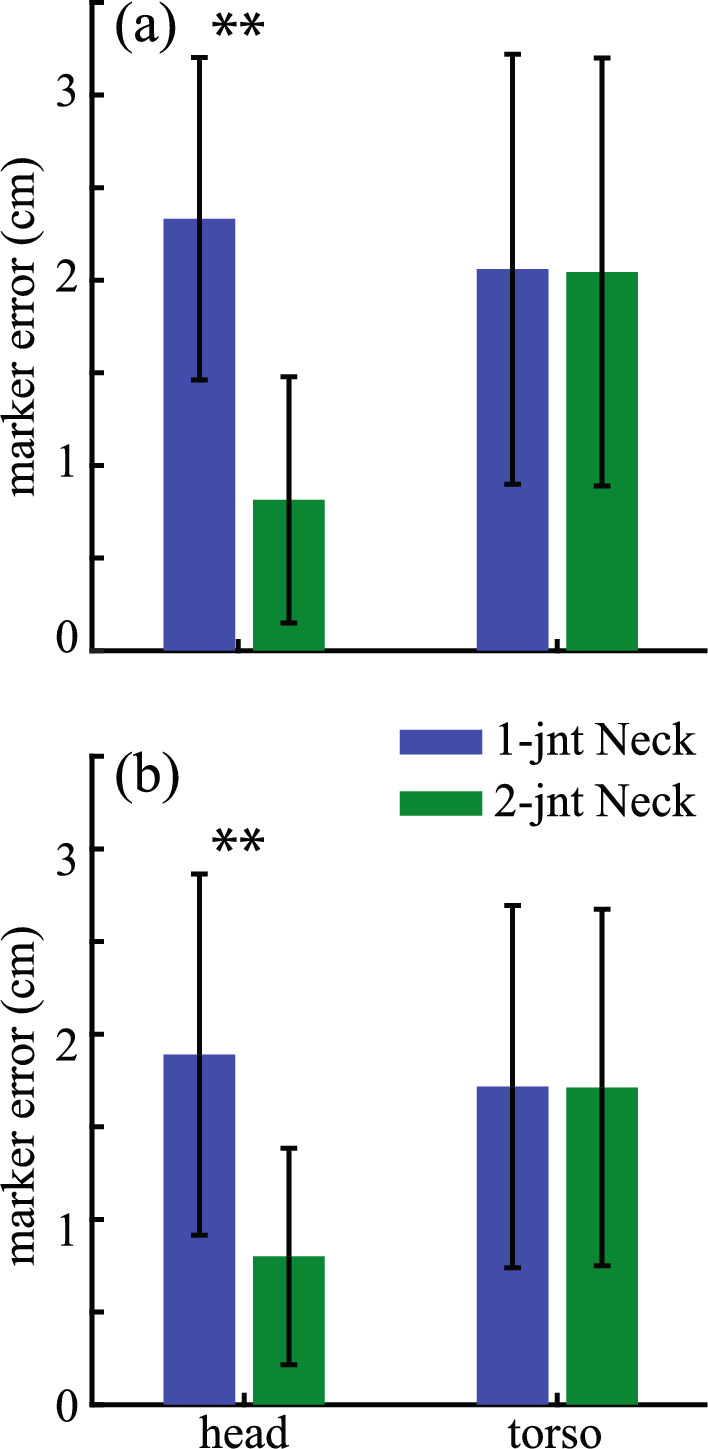
The comparisons of the tracking errors of the head and torso markers between two tested cervical spine models in **a** power and **b** speed athletes. Results shown are mean ± SD. **Indicates *p* < 0.001

When testing the inverse kinematic tracking of the markers on the head, the model with two-joint cervical spine performed better than the model with one-joint cervical spine. In both the speed athletes and power athletes, the model with two-joint cervical spine had the tracking errors of the head markers that were significantly lower than the model with one-joint cervical spine (Fig. 2; *p* < 0.001 for speed and power athletes). There was no difference in the tracking errors of the torso markers between the two cervical spine models (*p* = 0.34 for power athletes; *p* = 0.56 for speed athletes), which indicated that the torso segment was tracked well as the base segment during the testing of two cervical spine models.

**Fig. 3:**
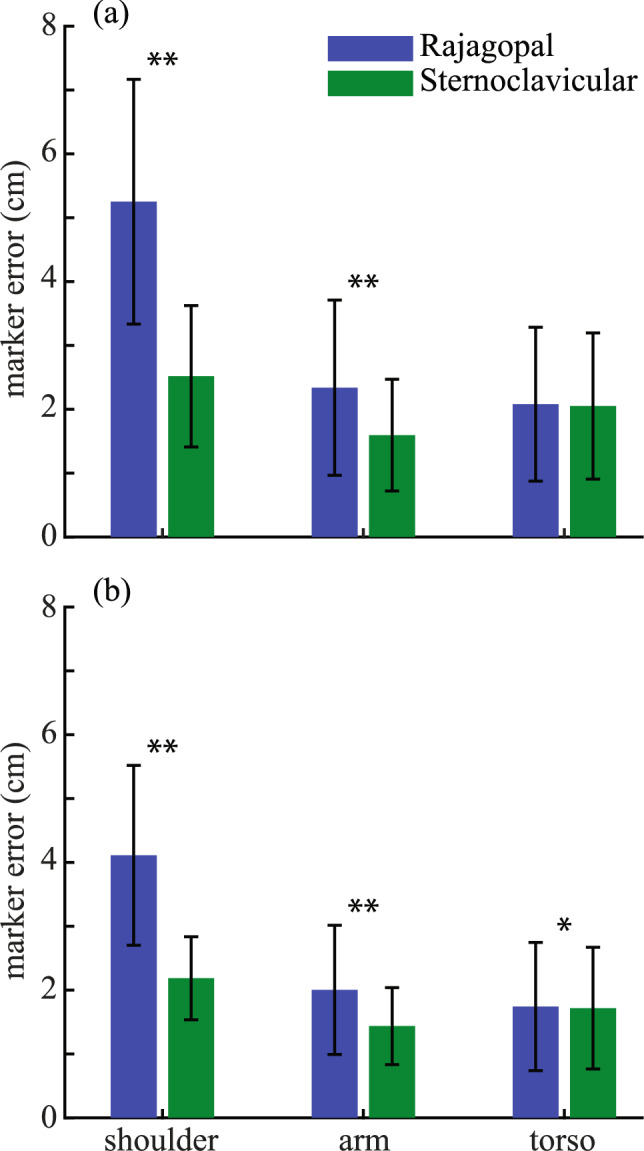
The comparisons of the tracking errors of the shoulder, arm and torso markers with and without (as in Ref. [19]) the sternoclavicular joint in **a** power and **b** speed athletes. Results shown are mean ± SD. *Indicates *p* < 0.05; **indicates *p* < 0.001

The addition of sternoclavicular joint greatly improved the tracking errors of the shoulder marker and even the arm markers. In both the speed and power athletes, the sternoclavicular joint significantly reduced the average tracking errors of the two shoulder markers (Fig. 3; *p* < 0.001 for speed and power athletes). The better tracking of the shoulder movement also led to significantly lower tracking errors of the arm markers (Fig. 3; *p* < 0.001 for speed and power athletes). In power athletes, there was no difference in the tracking errors of the torso markers (*p* = 0.16). Although there was a statistical difference in the tracking error of the torso markers in the speed athletes (*p* < 0.05), the actual magnitude of the difference is negligible 0.02 ± 0.05 cm. Thus, the torso segment was tracked well as the base segment during the testing of the effect of the added sternoclavicular joint.

**Fig. 4:**
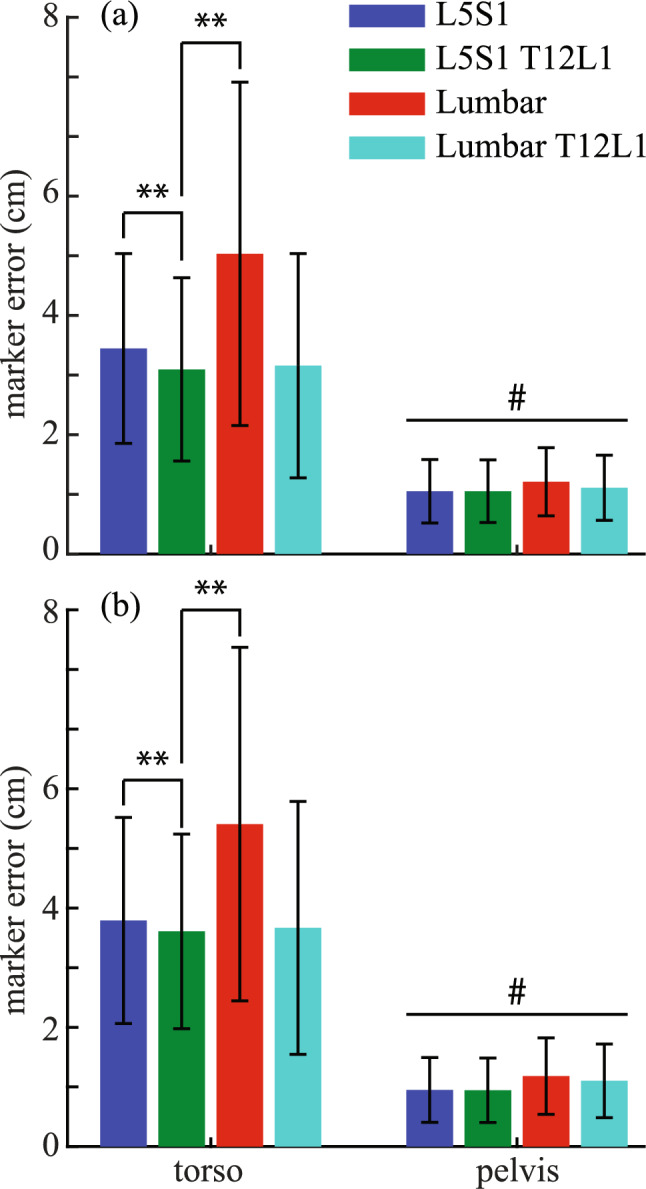
The comparisons of the tracking errors of the torso and pelvis markers with different representations of the spine in **a** power and **b** speed athletes. Results shown are mean ± SD. **Indicates *p* < 0.001. #Indicates model type as a significant fixed effect in marker tracking errors in pelvis at *p* < 0.001. However, no post hoc pairwise comparisons were performed because of small differences (~0.2 cm) in marker errors among various torso models

Of all tested torso models, the two-joint torso model had the lowest tracking errors of the torso markers. In both speed and power athletes, the average tracking errors of the five torso markers were significantly lower in the two-joint torso model than the one-joint torso model (Fig. 4; *p* < 0.001 for speed and power athletes) and the Lumbar model (*p* < 0.001 for speed and power athletes), and were comparable to the Lumbar-T12L1 model (*p* = 0.75 for speed athletes and *p* = 0.82 for power athletes). Interestingly, the detailed representation of the lumbar spine in the Lumbar model had an average tracking error of the five torso markers that was more than 1 cm higher than other torso models for both speed and power athletes. For both speed and power athletes, model type as a significant fixed effect existed on the average tracking errors of the four pelvis markers (Fig. 4; *p* < 0.001 for speed and power athletes). However, the actual differences were merely ~ 0.2 cm among various torso models that were tested, which indicated that the pelvis segment was tracked equally well for all four tested torso models. Therefore, no post hoc pairwise comparisons were performed.

**Fig. 5:**
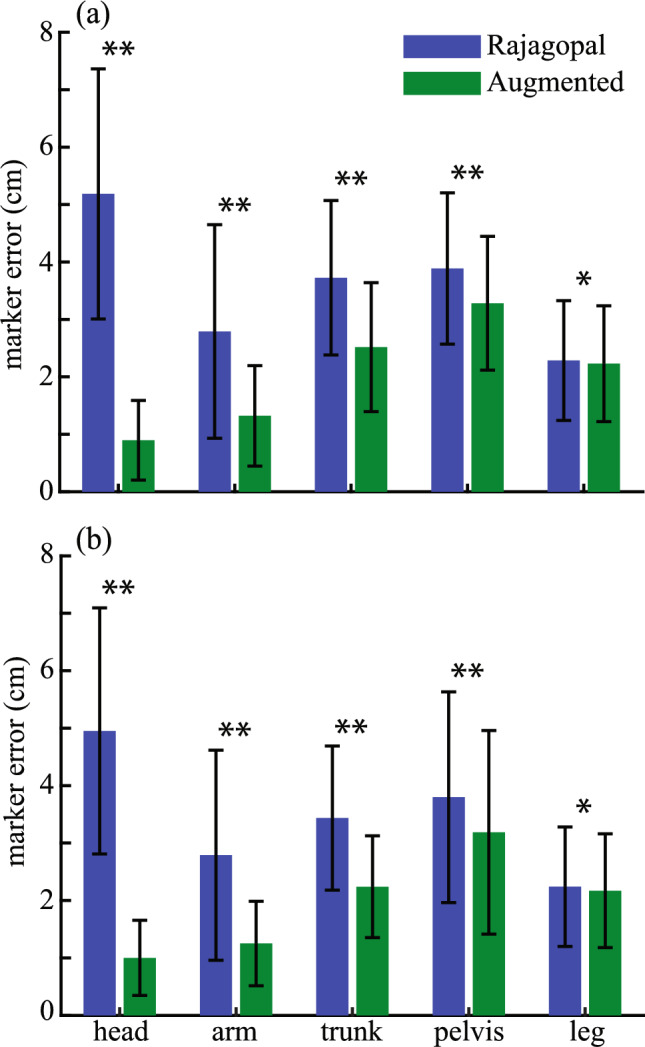
The comparison of the tracking errors of the markers from the head, arm, torso, pelvis, and leg segments with the original Rajagopal full-body model [19] and the augmented full-body model in **a** power and **b** speed athletes. Results shown are mean ± SD. *Indicates *p* < 0.05; **indicates *p* < 0.001

The augmented full-body model developed in this study performed significantly better than the original Rajagopal model [19] in tracking the movements during inverse kinematics. In the upper body, where the original model was augmented, for both speed and power athletes, the averages of the RMSE of the head markers, arm markers, and torso markers were significantly lower in the augmented full-body model than the original model (Fig. 5; all *p* < 0.001 for speed and power athletes). Although no augmentation was made below the pelvis, the tracking of the pelvis markers also improved in the augmented model compared to the original model (*p* < 0.001 for speed and power athletes). While there was significant improvement in the tracking of the leg markers in the speed and power athletes (*p* = 0.001 for speed athletes and *p* = 0.005 for power athletes), the improvement was merely 0.1 ± 0.1 cm on average across 30 leg markers for both types of athletes.

By better tracking the movements, the augmented full-body model also reduced the dynamic inconsistency during the inverse dynamics compared to the original Rajagopal model [19]. The average RMS ratios of three residual moments at the pelvis were all significantly below 1 (*p* < 0.001 for tilt, list, and rotation moments of pelvis). These ratios indicated about 20% reduction of residual moments at the pelvis in the augmented full-body model compared to the Rajagopal model. The average RMS ratios of the three residual forces at the pelvis were also significantly below 1 (*p* < 0.05 for anterior-posterior, vertical, and medial-lateral forces of pelvis). However, the actual reductions of the residual forces were about 3–10%, slightly smaller than the reduction in the residual moments.

## Discussion

The goal of this study was to augment the widely used Rajagopal full-body model [19] to improve the tracking of the kinematics of the head, neck, shoulder, and torso in activities that involve more complex upper body movements, such as the sports-related movements tested in this study. This was accomplished by adding five joints to connect the individual body segments that were split from the lumped upper body in the Rajagopal full-body model. Compared to the original Rajagopal full-body model, the augmented full-body model significantly improved the tracking of the sports-related movements that are more complex than typical walking during inverse kinematics, especially the upper body. The better tracking by the full-body model also led to reduced dynamic inconsistency between the model kinematics from inverse kinematics and the experimentally measured ground forces and moments. Together, these results indicated the improvement of the augmented full-body model compared to the Rajagopal model in both the kinematic and kinetic aspects. As the applications of musculoskeletal modeling and simulations have been greatly expanded beyond simple walking, the augmented model could be a more suitable model to perform simulations involving more complex body movements.

The augmentation of the original Rajagopal model [19] was based on a series of tests about various parts of the human upper body with the consideration of not substantially increasing the complexity of the model. To improve the tracking of the head movements, the two-joint neck model, which reflects possible independent motion of the upper and lower cervical spine [26], was added to the Rajagopal model because of the improved tracking of the head markers from this model over the 1-joint neck model (Fig. 2). This augmentation led to substantially improved tracking of the head markers when the augmented full-body model was tested against the Rajagopal model (Fig. 5), which does not include any joint in the cervical spine. The sternoclavicular joint was added to the Rajagopal model as a simplified shoulder girdle that connects the humerus to the torso. The protraction–retraction and elevation–depression of the clavicle enable the model to better track the shoulder marker compared to the Rajagopal model (Fig. 3). The better positioning of the shoulder resulted in improved tracking of the rest of the arm markers (Fig. 3). The two-joint torso model was used to represent the torso, since it improved the tracking of the torso markers while only minimally increasing the model’s complexity compared to other three tested models (Fig. 4). This augmentation of the torso improved the tracking of the torso markers compared to the Rajagopal model (Fig. 5), which only has one joint between L5 and S1 vertebrae. Overall, after all the augmentations were combined into the augmented full-body model, the tracking of the markers by the augmented model significantly improved across all upper body segments compared to the original Rajagopal full-body model (Fig. 5 and 7).

**Fig. 6:**
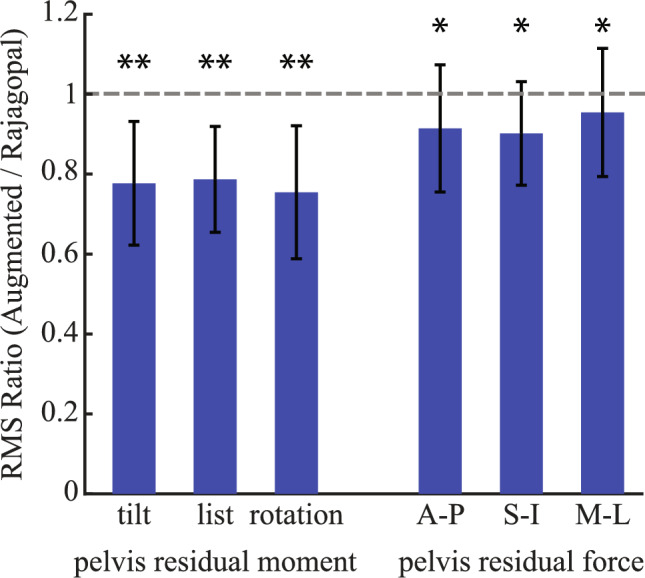
The comparison of the RMS ratio (augmented/Rajagopal full-body model) of the residual moments and forces from inverse dynamics. Results shown are mean ± SD. A–P: anterior-posterior; S–I: superior-inferior; M–L: medial-lateral. *Indicates ratios lower than 1 at *p* < 0.05; **indicates ratios lower than 1 at *p* < 0.001

The augmented full-body model not only offered better performance in inverse kinematics but also reduced the dynamic inconsistency in inverse dynamics, which is important in achieving high quality of dynamic simulations of movements that involve complex upper body movements. The reduced dynamic inconsistency demonstrated with the augmented full-body model was likely due to the model’s ability to better characterize the upper body motion involved in the 3 Cone Cut and Vcut movements, which more accurately represents the composite body center of mass. By contrast, the oversimplified upper body in the original Rajagopal model [19] may not be able to accurately represent the composite body center of mass when complex upper body motion was involved, contributing to increased dynamic inconsistency [35]. Indeed, the reduction of the residual moments was larger than those of residual forces (Fig. 6), which suggested the upper body kinematics was likely closer to the measured movements in the augmented model than the Rajagopal model, given identical total masses and modeling of the pelvis and lower limbs. Although the Residual Reduction algorithm [1] can be used to reduce dynamic inconsistency (i.e., the residual forces and moments), this is achieved by altering the mass parameters and the kinematics of the model. The reduced dynamic inconsistency with the augmented full-body model means less alterations to the model parameters and kinematics, so simulations that more closely reflect the experimentally recorded movements may be generated. The relative contribution to the reduced dynamic inconsistency from each of the added joints in the augmented model is likely motion dependent. As the added joints of the augmented model specifically aim to improve tracking of motion in head, torso, shoulder, and arm, their relative importance would be determined by to what extent each of these body segments is involved in the coordinated full-body motion to be assessed.

**Fig. 7:**
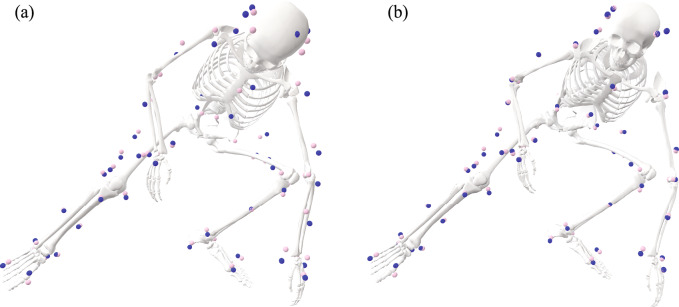
An example of the worse tracking of the upper body segments by **a** the original Rajagopal full-body model [19] than by **b** the augmented full-body model during inverse kinematics of one athlete performing the Agility movement. The same time frame of the same Agility movement was shown in (a) and (b)

The advantages in inverse kinematics and inverse dynamics of the augmented full-body model over the original Rajagopal model are likely movement dependent. In the sports-related movements tested in this study, the average RoMs of the DoFs added to the upper body of the augmented full-body model varied from 9.6 to 22.5 degrees, indicating substantial movements of individual upper body segments. Particularly, the average RoM of the lumbar flexion/extension was 22.5 ± 7.4 degrees, ranging from 37.3 to 59.8 degrees of flexion, and that of the lumbar bending (in frontal plane) was 18.0 ± 8.2 degrees, ranging from bending right 2.8 degrees to bending left 15.2 degrees. Not being able to account for the movements of various upper body segments was the main cause of the inferior performance of the original Rajagopal model compared to the augmented full-body model (Fig. 7). However, for simpler movements that involve subtle torso movement, such as walking, which involves less than 10 degrees RoMs in thoracic and lumbar spine [39], the original Rajagopal model should perform as well as the augmented full-body model.

The large torso flexion angles in the tested sports-related movements may have led to worse tracking performance from the torso model that incorporated the published lumbar spine model [21] than other torso models with simplified representation of spine (Fig. 4). In the lumbar model by Christophy, Senan [21], linear kinematic coordinate coupling constraints were used to distribute net torso flexion across the intervertebral joints in the lumbar spine based on the ratios reported in literature, such as [40] and [41]. These studies showed a decreasing trend of ratios from L1–L2 to L5–S1 intervertebral joints, which suggests that the upper portion of the lumbar spine contributes more to torso flexion than the lower portion. However, a recent review paper [42] suggested that the ratios of contribution actually have two distinct trends depending on the range of motion tested in the experiments: (1) a decreasing trend from upper to the lower portion of the lumbar spine when the tested flexion was under 45 degrees and (2) an increasing trend when the tested flexion was over 45 degrees. Although the lumbar model has been shown to benefit the kinematics tracking of the torso in jogging [43], which involves small torso flexions, it may not be suited for movements that involve large torso flexions, such as those encountered in our study, which had average torso flexion angles at 52.4 ± 12.2 degrees as estimated in IK with the one-joint torso model.

Although the augmented full-body model developed in this study demonstrated improved performance in inverse kinematics and inverse dynamics compared to the Rajagopal full-body model [19], it has its own limitations. In the augmented full-body model, the shoulder portion of the model is represented by the 2-DoF sternoclavicular joint with the scapula welded to the clavicle at the acromioclavicular joint. We simplified the shoulder girdle this way to capture the motion primarily originated from the protraction–retraction and elevation–depression of the clavicle, which is important to properly position the glenohumeral joint to track arm in complex full-body motion, while not increasing the complexity of the model. With this simplification, the model may not be able to accurately describe the motion of the scapula. More complex upper limb or shoulder models involving shoulder rhythm [29, 44, 45] need to be incorporated to fully describe the shoulder motion. However, since the motion tracked in the study involved all major body segments in the lower and upper body, not just focusing on the scapula, the overall results and conclusions likely were not affected by the simplification of the shoulder. In addition, the augmented full-body model uses two joints in the lumbar and thoracic spine to characterize the torso movements, which limits its capability of assessing spinal loadings. There are full-body models with fully articulated spines [20, 24, 46], which are developed for estimating spinal loadings. However, this capability requires the drastic increase of the complexity of the torso model to have over 50 DoFs. Since the purpose of the augmented full-body model was to improve the overall tracking performance in inverse kinematics rather than assessing spinal loadings, the simplified two-joint torso model was used.

In conclusion, as the applications of musculoskeletal simulations are expanding to more complex movements involved in various types of sports in addition to simple walking, a musculoskeletal model that can characterize the movements of not only the lower limbs but also the segments in the upper body is necessary to develop high-quality simulations which are dynamically consistent with the recorded motion. Building upon the original Rajagopal full-body model [19], the augmented full-body model developed in this study improved the tracking of the upper body segments in inverse kinematics and reduced dynamic inconsistency in inverse dynamics without increasing the overall complexity of the model and the number of markers needed in motion capture. Since lower limb injuries are the most common injuries in sports [17] and appropriate modeling of upper body motion is important to accurately estimate lower limb loadings [23], we hope that this augmented model can help research groups in the field to generate dynamic simulations that advance our understanding of the biomechanics and injury mechanisms of the lower limbs across various sports.
